# The FGF23/α-Klotho Axis in Postmenopausal Osteoporosis: Associations with Bone Mineral Density and Diagnostic Discrimination

**DOI:** 10.3390/jcm15176716

**Published:** 2026-08-29

**Authors:** Mete Hakan Karalok, Naile Fevziye Misirlioglu, Oznur Dundar Akin, Hafize Uzun

**Affiliations:** 1 Department of Obstetrics and Gynecology, Faculty of Medicine, Istanbul Atlas University, Istanbul 34408, Türkiye; oznur.akin@atlas.edu.tr; 2 Department of Biochemistry, Faculty of Medicine, Istanbul Nişantaşı University, Istanbul 34398, Türkiye; nailemisirlioglu@gmail.com; 3 Department of Biochemistry, Faculty of Medicine, Istanbul Atlas University, Istanbul 34408, Türkiye; hafize.uzun@atlas.edu.tr

**Keywords:** osteoporosis, fibroblast growth factor 23, α-Klotho, bone mineral density, biomarker, ROC analysis, postmenopausal women

## Abstract

**Background:** Fibroblast growth factor 23 (FGF23) and α-Klotho are key regulators of mineral metabolism and bone homeostasis; however, their combined diagnostic value in postmenopausal osteoporosis remains incompletely understood. This study investigated the associations of serum FGF23, α-Klotho, and the FGF23/α-Klotho ratio with bone mineral density (BMD), bone turnover markers, and their diagnostic performance for postmenopausal osteoporosis. **Methods:** This cross-sectional study included 165 women divided into three groups: premenopausal healthy controls (n = 55), postmenopausal non-osteoporotic women (n = 55), and postmenopausal women with osteoporosis (n = 55). Serum FGF23 and α-Klotho concentrations were measured by enzyme-linked immunosorbent assay. Bone mineral density was assessed by dual-energy X-ray absorptiometry. Correlation analyses, age- and BMI-adjusted partial correlations, multivariable linear and logistic regression analyses, receiver operating characteristic (ROC) analyses, and incremental diagnostic models were performed. Diagnostic ROC and incremental model analyses were restricted to postmenopausal women (n = 110). **Results:** Serum FGF23 concentrations and the FGF23/α-Klotho ratio increased progressively across the study groups, whereas α-Klotho levels decreased (all *p* < 0.001). FGF23 was inversely correlated with lumbar spine, femoral neck, and total hip BMD and T-scores (all *p* < 0.001), whereas α-Klotho demonstrated positive correlations with all BMD parameters (all *p* < 0.001). These associations remained significant after adjustment for age and BMI. In multivariable linear regression analyses, the FGF23/α-Klotho ratio showed the strongest independent association with skeletal T-scores (standardized β = −0.379 to −0.404; all *p* < 0.001). Multivariable logistic regression identified lower 25-hydroxyvitamin D, higher parathyroid hormone, higher FGF23, and lower α-Klotho as independent factors associated with osteoporosis. In analyses restricted to postmenopausal women, the FGF23/α-Klotho ratio showed the highest numerical discriminative performance for osteoporosis (AUC = 0.832, 95% CI: 0.755–0.901), followed by α-Klotho (AUC = 0.793) and FGF23 (AUC = 0.697). The ratio significantly outperformed FGF23 alone (ΔAUC = 0.135, *p* = 0.001), whereas its performance did not differ significantly from that of α-Klotho (ΔAUC = 0.038, *p* = 0.270). Adding FGF23 and α-Klotho to age, BMI, and 25-hydroxyvitamin D increased the AUC from 0.743 to 0.882. **Conclusions:** Serum FGF23 and α-Klotho are independently associated with bone mineral density and are linked to bone turnover markers in postmenopausal women. The FGF23/α-Klotho ratio showed the highest numerical discriminative performance among the evaluated biomarkers, although its performance was not significantly different from that of α-Klotho alone. The combined assessment of FGF23 and α-Klotho provided additional discriminatory information beyond the measured clinical variables. Given the cross-sectional design and lack of external validation, these findings should be considered exploratory and require validation in prospective independent cohorts.

## 1. Introduction

Postmenopausal osteoporosis (PMOP) is a systemic metabolic bone disorder characterized by progressive loss of bone mass and deterioration of bone microarchitecture, leading to increased bone fragility and a substantially higher risk of osteoporotic fractures [1]. The decline in estrogen after menopause accelerates bone remodeling and shifts the balance toward bone resorption, leading to progressive loss of trabecular and cortical bone. Although dual-energy X-ray absorptiometry (DXA)-derived bone mineral density (BMD) remains the standard approach for diagnosing osteoporosis and estimating fracture risk, BMD alone does not fully capture the dynamic biological processes underlying bone loss [1,2,3]. Therefore, biochemical markers reflecting mineral metabolism, bone turnover, and endocrine regulation may provide additional insight into the pathophysiology of PMOP.

Bone remodeling is regulated by a complex interaction among calcium–phosphate homeostasis, parathyroid hormone (PTH), vitamin D, renal function, and osteoblast–osteoclast activity. Bone turnover markers such as procollagen type I N-terminal propeptide (P1NP), osteocalcin, alkaline phosphatase, and β-C-terminal telopeptide of type I collagen (β-CTX) are widely used to reflect bone formation and resorption dynamics [4,5,6,7]. In postmenopausal women, increased bone turnover is associated with accelerated bone loss and may contribute to skeletal fragility beyond what is explained by BMD alone [8,9]. However, the endocrine mediators linking mineral metabolism to bone turnover and osteoporosis risk remain incompletely clarified.

Fibroblast growth factor 23 (FGF23) is a bone-derived hormone mainly produced by osteocytes and osteoblasts. It plays a central role in phosphate and vitamin D metabolism by promoting renal phosphate excretion and suppressing 1,25-dihydroxyvitamin D synthesis [10,11]. The biological actions of FGF23 are largely mediated through fibroblast growth factor receptors in the presence of α-Klotho, a transmembrane and soluble protein that functions as an essential co-receptor and is also recognized as an anti-aging molecule [12,13]. Experimental and clinical evidence suggests that dysregulation of the FGF23/α-Klotho axis may contribute not only to mineral metabolism abnormalities but also to altered bone remodeling, impaired mineralization, and increased fracture susceptibility [14,15].

Previous studies evaluating the relationship between FGF23, α-Klotho, and skeletal health have reported heterogeneous findings. Some clinical studies have shown that higher FGF23 levels are associated with increased bone turnover or lower BMD in postmenopausal osteoporosis, whereas other population-based studies have reported weak or non-significant associations [16,17,18]. Similarly, α-Klotho has been linked to BMD and osteoporosis risk in postmenopausal women, but the direction and magnitude of these associations vary across populations and study designs [13,19,20,21]. These inconsistencies may partly reflect differences in age, renal function, menopausal status, vitamin D status, and the use of single biomarkers rather than integrated measures of the FGF23/α-Klotho axis.

The FGF23/α-Klotho ratio may provide a more comprehensive reflection of the functional balance between FGF23 activity and Klotho availability than either biomarker alone. Nevertheless, data on the diagnostic and clinical relevance of this ratio in postmenopausal osteoporosis remain limited. Few studies have simultaneously evaluated serum FGF23, α-Klotho, their ratio, DXA-derived BMD, bone turnover markers, and diagnostic performance using receiver operating characteristic analyses.

Therefore, this study aimed to investigate serum FGF23, α-Klotho, and the FGF23/α-Klotho ratio in premenopausal healthy women, postmenopausal non-osteoporotic women, and postmenopausal women with osteoporosis. We further examined their associations with BMD, bone turnover markers, mineral metabolism parameters, and their discriminative performance for identifying osteoporosis among postmenopausal women.

## 2. Materials and Methods

### 2.1. Study Design and Ethical Approval

This cross-sectional study was conducted in accordance with the ethical principles of the Declaration of Helsinki and was approved by the Istanbul Atlas University Health Sciences Scientific Research Ethics Committee (approval date: 16 June 2026; approval no. E-22686390-050.99-102081). All participants were informed in detail about the study objectives and procedures prior to participation, and electronic informed consent was obtained.

#### Study Population

This cross-sectional study was conducted at the Department of Obstetrics and Gynecology, Istanbul Atlas University Hospital. Eligible women attending the department were consecutively screened according to the predefined inclusion and exclusion criteria and were invited to participate in the study. Women who fulfilled the eligibility criteria and provided informed consent were enrolled. Characteristics of eligible women who declined participation were not systematically recorded; therefore, a formal comparison between participants and non-participants could not be performed. A total of 165 women were enrolled and classified into three equal groups according to menopausal status and BMD: premenopausal healthy women (n = 55), postmenopausal non-osteoporotic women (n = 55), and postmenopausal women with osteoporosis (n = 55). Postmenopausal women were considered osteoporotic if they had undergone natural menopause (absence of menstruation for ≥12 months) and met the WHO densitometric criterion for osteoporosis, defined as a T-score ≤ −2.5 at the lumbar spine, femoral neck, or total hip, as determined by dual-energy X-ray absorptiometry (DXA) [22]. Postmenopausal women with T-scores > −2.5 at all evaluated skeletal sites were classified as the postmenopausal non-osteoporotic group. Group allocation was based on the prespecified DXA-based operational definition. Eighteen participants had a documented history of fragility fracture. Among the postmenopausal women, six participants with a history of fragility fracture had T-scores > −2.5 at all evaluated skeletal sites and were therefore classified into the postmenopausal non-osteoporotic group according to the study’s DXA-based definition. Fragility fracture history was not used as an independent criterion for group assignment.

### 2.2. Inclusion and Exclusion Criteria

#### 2.2.1. Inclusion Criteria

Women aged 40 years or older who were able to provide informed consent were eligible for inclusion. Participants were assigned to the study groups according to menopausal status and DXA findings.

#### 2.2.2. Exclusion Criteria

Women with diseases or conditions known to influence bone metabolism were excluded, including secondary osteoporosis, chronic kidney disease, chronic liver disease, malignancy, inflammatory or autoimmune diseases, thyroid or parathyroid disorders, uncontrolled endocrine diseases, pregnancy or lactation, and acute infection. Medication use was reviewed as part of the eligibility assessment. Individuals receiving medications affecting bone metabolism, including systemic glucocorticoids, antiresorptive agents, anabolic osteoporosis treatments, or hormone replacement therapy, were also excluded. Use of other medications not considered to have a major effect on bone or mineral metabolism did not constitute an exclusion criterion; however, detailed indication-level information on these concomitant medications was not systematically collected.

### 2.3. Clinical Evaluation and Bone Mineral Density Measurements

Age, body weight, height, and body mass index (BMI) were recorded for all participants. Bone mineral density (BMD) was measured at the lumbar spine (L1–L4), femoral neck, and total hip using dual-energy X-ray absorptiometry (DXA) (Hologic Inc., Bedford, MA, USA). BMD values were expressed as g/cm^2^, and corresponding T-scores were calculated according to the manufacturer’s reference database. Participants were classified into study groups based on these measurements.

### 2.4. Data Collection and Laboratory Measurements

#### 2.4.1. Sample Collection and Biochemical Parameters

Venous blood samples were collected between 08:00 and 10:00 a.m. after an overnight fast of at least 8 h. Serum samples were separated by centrifugation at 3000 rpm for 10 min, aliquoted into sterile tubes, and stored at −80 °C until analysis.

Routine laboratory measurements, including serum calcium, corrected calcium, phosphorus, magnesium, alkaline phosphatase (ALP), parathyroid hormone (PTH), 25-hydroxyvitamin D [25(OH)D], procollagen type I N-terminal propeptide (P1NP), β-C-terminal telopeptide of type I collagen (β-CTX), urea, creatinine, estimated glomerular filtration rate (eGFR), and C-reactive protein (CRP), were performed according to the manufacturer’s instructions using a Cobas e601 immunoassay analyzer (Roche Diagnostics, Basel, Switzerland).

#### 2.4.2. Measurement of Serum Fibroblast Growth Factor 23 (FGF23) and α-Klotho Levels

Serum FGF23 concentrations were measured using a commercially available human sandwich enzyme-linked immunosorbent assay (ELISA) kit (Cloud-Clone Corp., Katy, TX, USA; Catalog No. SEA746Hu) according to the manufacturer’s instructions. The assay had a detection range of 15.6–1000 pg/mL, a minimum detectable concentration of <6.2 pg/mL, an intra-assay coefficient of variation (CV) < 10%, and an inter-assay CV < 12%.

Serum α-Klotho concentrations were determined using a commercially available Human Klotho (KL) ELISA Kit (Elabscience Biotechnology Inc., Houston, TX, USA; Catalog No. E-EL-H5451) according to the manufacturer’s protocol. The assay exhibited a detection range of 0.31–20 ng/mL, a sensitivity of 0.19 ng/mL, and an intra- and inter-assay CV < 10%.

All serum samples were analyzed in duplicate, and the mean value of the duplicate measurements was used for statistical analyses. Optical density was measured at 450 nm using a microplate reader (Thermo Fisher Scientific, Waltham, MA, USA), and analyte concentrations were calculated from standard curves generated for each assay according to the manufacturers’ protocols.

For calculation of the FGF23/α-Klotho ratio, α-Klotho concentrations were converted to pg/mL to ensure unit consistency. The ratio was then calculated for each participant as serum FGF23 (pg/mL) divided by serum α-Klotho (pg/mL), using the raw assay-derived concentrations. No standardization, cross-assay normalization, or mathematical transformation was applied before calculation of the primary ratio. The ratio was regarded as an exploratory composite index rather than a stoichiometric measure of FGF23–Klotho signaling.

### 2.5. Statistical Analysis

Statistical analyses were performed using IBM SPSS Statistics version 31.0 (IBM Corp., Armonk, NY, USA) and MedCalc Statistical Software version 22.0 (MedCalc Software Ltd., Ostend, Belgium). The distribution of continuous variables was assessed using the Shapiro–Wilk test. Continuous variables are presented as mean ± standard deviation (SD), whereas categorical variables are expressed as numbers and percentages. Comparisons among the three groups were performed using one-way analysis of variance (ANOVA), followed by Tukey’s honestly significant difference (HSD) post hoc test for pairwise comparisons. Categorical variables were compared using the chi-square test. Spearman’s rank correlation analysis was used to evaluate associations between serum FGF23, α-Klotho, and clinical or biochemical variables. Serum FGF23 and α-Klotho concentrations showed no substantial deviation from normality, whereas the FGF23/α-Klotho ratio exhibited right-skewness. Accordingly, rank-based correlation analyses were used for association analyses. To evaluate the robustness of ratio-based findings, sensitivity analyses were additionally performed using the natural logarithm of the FGF23/α-Klotho ratio. Alternative joint modeling was also evaluated by entering serum FGF23 and α-Klotho simultaneously as separate variables in the multivariable logistic and incremental diagnostic models. Age- and BMI-adjusted partial Spearman correlation analyses were additionally performed. Multivariable linear regression analyses were conducted to identify factors independently associated with bone mineral density, whereas multivariable binary logistic regression analysis was used to determine factors independently associated with osteoporosis. Multivariable binary logistic regression analyses addressing postmenopausal osteoporosis were restricted to postmenopausal participants (n = 110).

Receiver operating characteristic (ROC) curve analysis was performed to assess the diagnostic performance of serum FGF23, α-Klotho, and the FGF23/α-Klotho ratio. For analyses evaluating the diagnostic performance for postmenopausal osteoporosis, ROC analyses and incremental diagnostic models were restricted to postmenopausal participants, comparing postmenopausal women with osteoporosis with postmenopausal non-osteoporotic women (n = 110). Premenopausal healthy participants were not included in these diagnostic analyses. The area under the ROC curve (AUC), 95% confidence intervals (CIs), optimal cut-off values determined by the Youden index, sensitivity, and specificity were calculated. Pairwise comparisons of AUCs were performed using paired bootstrap resampling with 2000 iterations. Sequential ROC models were constructed to evaluate the incremental diagnostic value of FGF23 and α-Klotho beyond conventional clinical risk factors. Incremental differences in AUC between nested diagnostic models were formally evaluated using paired bootstrap resampling with 2000 iterations, and 95% CIs for ΔAUC were calculated. For the final combined biomarker model versus the clinical reference model, category-free net reclassification improvement (NRI) and integrated discrimination improvement (IDI) were additionally calculated with bootstrap 95% CIs. The final sequential model was separately internally validated using 2000 bootstrap resamples to estimate optimism-corrected AUC and Brier score, calibration slope, and calibration intercept. A calibration plot of the final sequential model was additionally generated and is presented as Supplementary Figure S1.

Given the established relationship between renal function and the FGF23–Klotho axis, additional sensitivity diagnostic models were constructed by adding eGFR to the clinical reference model. The eGFR-adjusted reference model included age, BMI, 25-hydroxyvitamin D, and eGFR, after which FGF23, α-Klotho, the FGF23/α-Klotho ratio, or FGF23 and α-Klotho jointly were added. Differences in AUC relative to the eGFR-adjusted reference model were evaluated using paired bootstrap resampling with 2000 iterations.

To evaluate the robustness of the DXA-based group classification, a sensitivity analysis was additionally performed after excluding six postmenopausal women with a history of fragility fracture who had T-scores > −2.5 at all evaluated skeletal sites. The principal three-group comparisons, age- and BMI-adjusted partial correlation analyses, and multivariable linear regression analyses were repeated in the remaining 159 participants. For the multivariable logistic regression, diagnostic ROC, and incremental model sensitivity analyses, these six women were excluded from the postmenopausal cohort, resulting in 104 postmenopausal participants (49 non-osteoporotic and 55 osteoporotic women).

Internal validation of the multivariable logistic regression model for postmenopausal osteoporosis was performed using bootstrap resampling with 2000 iterations. In each bootstrap sample, the complete model was refitted, and its performance was evaluated both in the bootstrap sample and in the original dataset to estimate optimism. Model discrimination was assessed using the AUC, whereas calibration was evaluated using the calibration slope, calibration-in-the-large intercept, and Brier score. Optimism-corrected performance estimates were subsequently calculated. A calibration plot comparing predicted probabilities with observed outcome frequencies was also generated.

An a priori sample-size calculation was performed using G*Power version 3.1. The calculation was based on the primary comparison among the three independent study groups using one-way ANOVA. Assuming a medium effect size (Cohen’s *f* = 0.25), a two-sided α level of 0.05, and 80% statistical power, approximately 159 participants were required. To account for possible exclusions, incomplete data, or withdrawal, the target sample size was increased to 55 participants per group, resulting in a planned total sample size of 165 participants. The primary outcome for sample-size determination was the between-group difference in serum FGF23 and α-Klotho concentrations across the three study groups. All statistical tests were two-tailed, and a *p* value < 0.05 was considered statistically significant.

## 3. Results

Table 1 summarizes the baseline demographic characteristics of the study population. Significant differences in age were observed among the three groups (*p* < 0.001), with both postmenopausal groups being older than the premenopausal healthy controls. Body mass index also differed significantly across the groups (*p* = 0.014). Among postmenopausal women, the osteoporotic group was a significantly lower age at menopause than the non-osteoporotic group (*p* = 0.044), whereas menopause duration was comparable between the two postmenopausal groups (*p* = 0.509). No significant differences were observed among the groups regarding smoking status, alcohol consumption, physical activity level, or family history of osteoporosis (all *p* > 0.05).

Biochemical characteristics are summarized in Table 2. Serum phosphorus, ALP, bone-specific ALP, PTH, 25-hydroxyvitamin D, osteocalcin, P1NP, β-CTX, eGFR, and CRP differed significantly among the three groups (all *p* ≤ 0.002). Bone turnover markers, including bone-specific ALP, osteocalcin, P1NP, and β-CTX, as well as PTH, increased progressively from premenopausal healthy women to postmenopausal osteoporotic women, whereas 25-hydroxyvitamin D and eGFR showed a progressive decline. Post hoc Tukey HSD analysis confirmed significant differences in most parameters, with the highest bone turnover marker levels and lowest vitamin D and eGFR values observed in the osteoporotic group. Serum calcium, corrected calcium, magnesium, urea, and creatinine did not differ significantly among the groups (all *p* > 0.05).

**Figure 1:**
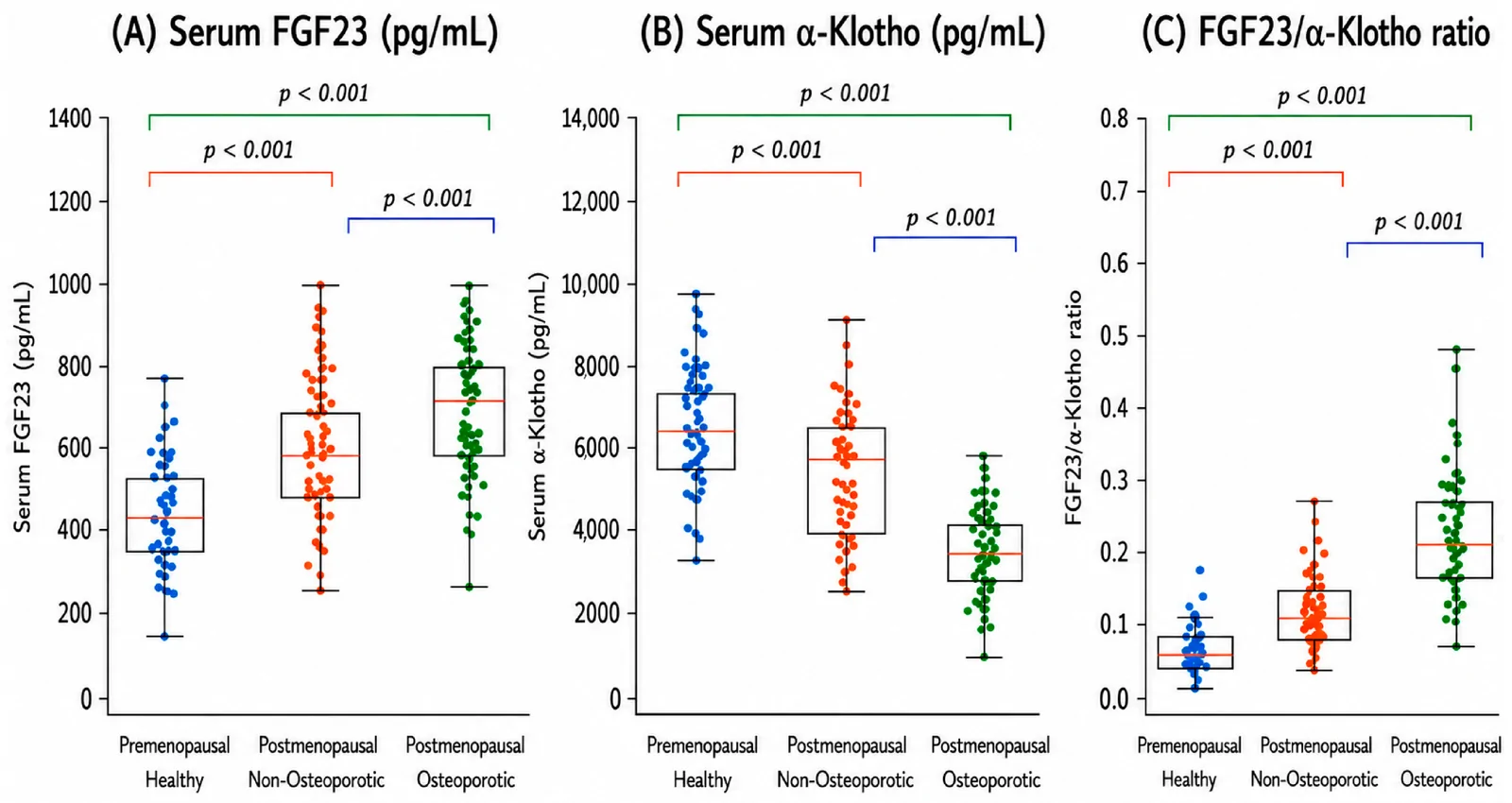
Distribution of serum FGF23 (**A**), α-Klotho (**B**), and the FGF23/α-Klotho ratio (**C**) according to study groups. Box plots represent the median and interquartile range, with whiskers indicating the minimum and maximum values. Individual data points are shown. Pairwise comparisons were performed using Tukey’s honestly significant difference (HSD) post hoc test; all comparisons were statistically significant (*p* < 0.001).

Figure 1 illustrates the distributions of serum FGF23, α-Klotho, and the FGF23/α-Klotho ratio across the study groups. Serum FGF23 concentrations increased progressively from premenopausal healthy women to postmenopausal non-osteoporotic women and further to postmenopausal osteoporotic women, whereas serum α-Klotho levels showed the opposite trend, with a progressive decrease across the three groups. Consequently, the FGF23/α-Klotho ratio increased significantly from the premenopausal healthy group to the postmenopausal non-osteoporotic group and reached the highest levels in postmenopausal osteoporotic women. Post hoc pairwise comparisons demonstrated significant differences between all three groups for serum FGF23, α-Klotho, and the FGF23/α-Klotho ratio (all adjusted *p* < 0.001).

**Figure 2:**
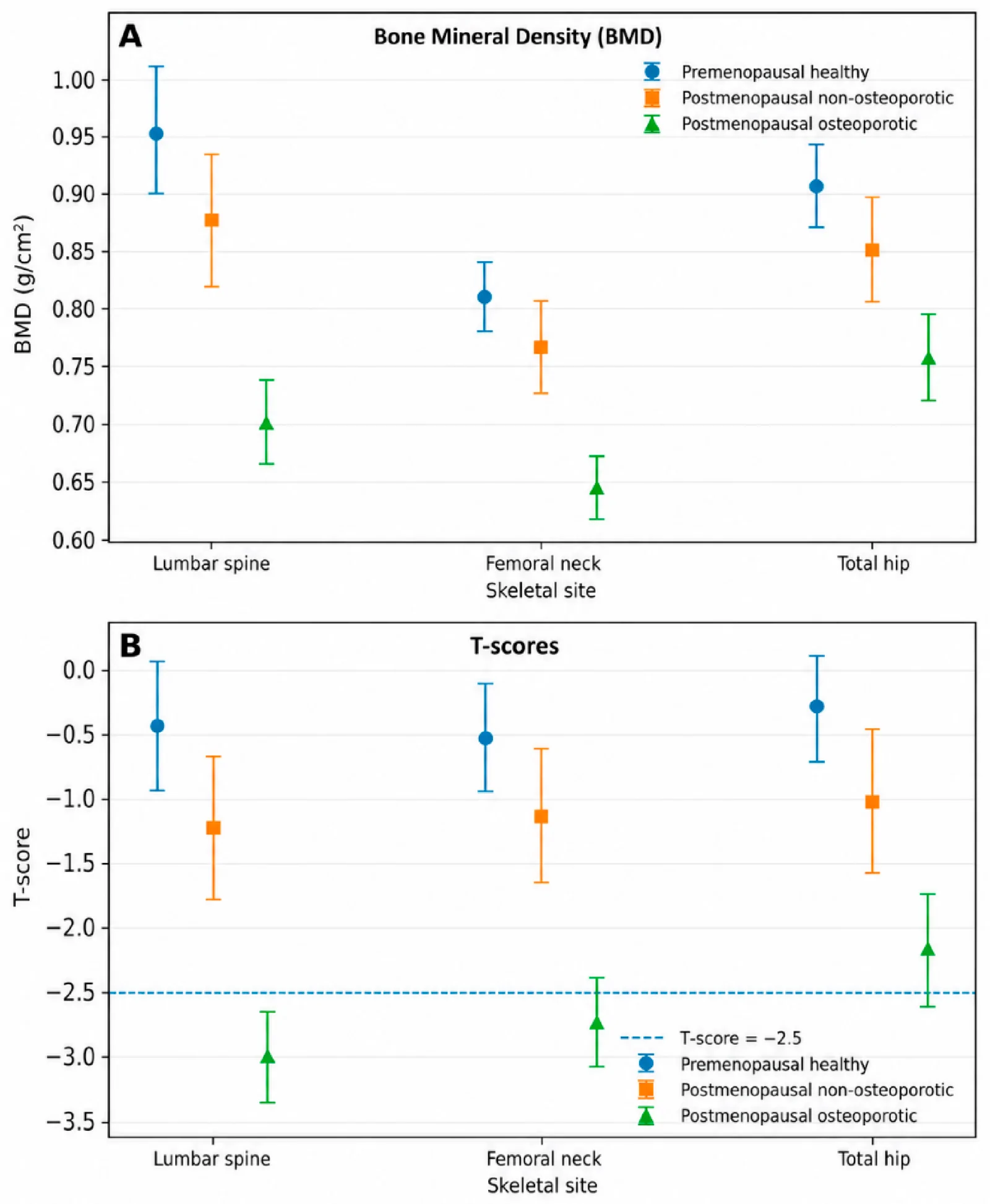
Bone mineral density and T-score measurements according to study groups. (**A**) Mean bone mineral density (BMD) values at the lumbar spine, femoral neck, and total hip. (**B**) Mean T-scores at the corresponding skeletal sites. Data are presented as mean ± standard deviation. The dashed horizontal line in panel B indicates the diagnostic T-score threshold of −2.5. Premenopausal healthy women showed the highest BMD and T-score values, whereas postmenopausal women with osteoporosis exhibited the lowest values at all skeletal sites.

Bone mineral density parameters differed significantly among the three study groups (Figure 2 and Supplementary Table S1). Premenopausal healthy women had the highest BMD and T-score values at all skeletal sites, whereas postmenopausal osteoporotic women exhibited the lowest values (all *p* < 0.001). Tukey HSD post hoc analysis confirmed significant pairwise differences among all three groups for lumbar spine, femoral neck, and total hip BMD and T-score measurements (all adjusted *p* < 0.001), demonstrating a progressive decline in bone mineral density across the three study groups.

Table 3 presents the Spearman correlation analysis between serum FGF23, α-Klotho, bone mineral density, and biochemical parameters. Serum FGF23 was significantly and inversely correlated with lumbar spine, femoral neck, and total hip BMD and T-scores (all r = −0.487 to −0.509, all *p* < 0.001), whereas serum α-Klotho showed significant positive correlations with all BMD parameters (r = 0.452–0.481, all *p* < 0.001). Furthermore, FGF23 was positively correlated with phosphorus, ALP, bone-specific ALP, PTH, osteocalcin, P1NP, and β-CTX, while showing negative correlations with 25-hydroxyvitamin D and eGFR (all *p* < 0.05). In contrast, α-Klotho demonstrated the opposite correlation pattern, exhibiting negative correlations with phosphorus, bone turnover markers, and PTH, and positive correlations with 25-hydroxyvitamin D and eGFR (all *p* < 0.05).

Table 4 presents the age- and BMI-adjusted partial Spearman correlation analysis between serum FGF23, α-Klotho, the FGF23/α-Klotho ratio, and bone mineral density parameters. After adjustment for age and BMI, serum FGF23 remained significantly and inversely correlated with lumbar spine, femoral neck, and total hip T-scores (ρ = −0.326 to −0.346, all *p* < 0.001). In contrast, serum α-Klotho remained positively correlated with all BMD parameters (ρ = 0.455–0.522, all *p* < 0.001). The FGF23/α-Klotho ratio exhibited the strongest inverse correlations with lumbar spine, femoral neck, and total hip T-scores (ρ = −0.504 to −0.544, all *p* < 0.001), indicating that these associations were independent of age and BMI.

Table 5 summarizes the multivariable linear regression analyses of factors associated with bone mineral density T-scores. After adjustment for age, BMI, PTH, 25-hydroxyvitamin D, and eGFR, serum FGF23 remained independently and inversely associated with lumbar spine, femoral neck, and total hip T-scores (standardized β = −0.227 to −0.247, all *p* < 0.001). Conversely, serum α-Klotho was independently and positively associated with all BMD parameters (standardized β = 0.327–0.380, all *p* < 0.001). Among the evaluated biomarkers, the FGF23/α-Klotho ratio showed the strongest independent inverse association with bone mineral density (standardized β = −0.379 to −0.404, all *p* < 0.001), with the highest adjusted R^2^ values across all regression models (0.548–0.602).

Because the FGF23/α-Klotho ratio showed right-skewness, the ratio-based multivariable analyses were repeated using its natural-log transformation. The findings remained consistent, with significant inverse associations with lumbar spine (standardized β = −0.433), femoral neck (β = −0.373), and total hip T-scores (β = −0.411; all *p* < 0.001). ROC discrimination was unchanged (AUC = 0.832).

Table 6 presents the multivariable logistic regression analysis restricted to postmenopausal women (n = 110). After adjustment for age, BMI, 25-hydroxyvitamin D, PTH, FGF23, and α-Klotho, lower serum 25-hydroxyvitamin D levels (OR = 0.83, 95% CI: 0.73–0.94, *p* = 0.003), higher PTH levels (OR = 1.11, 95% CI: 1.05–1.16, *p* < 0.001), higher serum FGF23 concentrations (OR = 1.007, 95% CI: 1.003–1.012, *p* = 0.002), and lower α-Klotho concentrations (OR = 0.9985, 95% CI: 0.9977–0.9993, *p* < 0.001) were independently associated with postmenopausal osteoporosis. Age showed an inverse association with osteoporosis (OR = 0.72, 95% CI: 0.53–0.96, *p* = 0.028), whereas BMI was not independently associated with osteoporosis (*p* = 0.354). The overall model was statistically significant (likelihood ratio test, *p* < 0.001) and showed a Nagelkerke R^2^ of 0.759.

**Figure 3:**
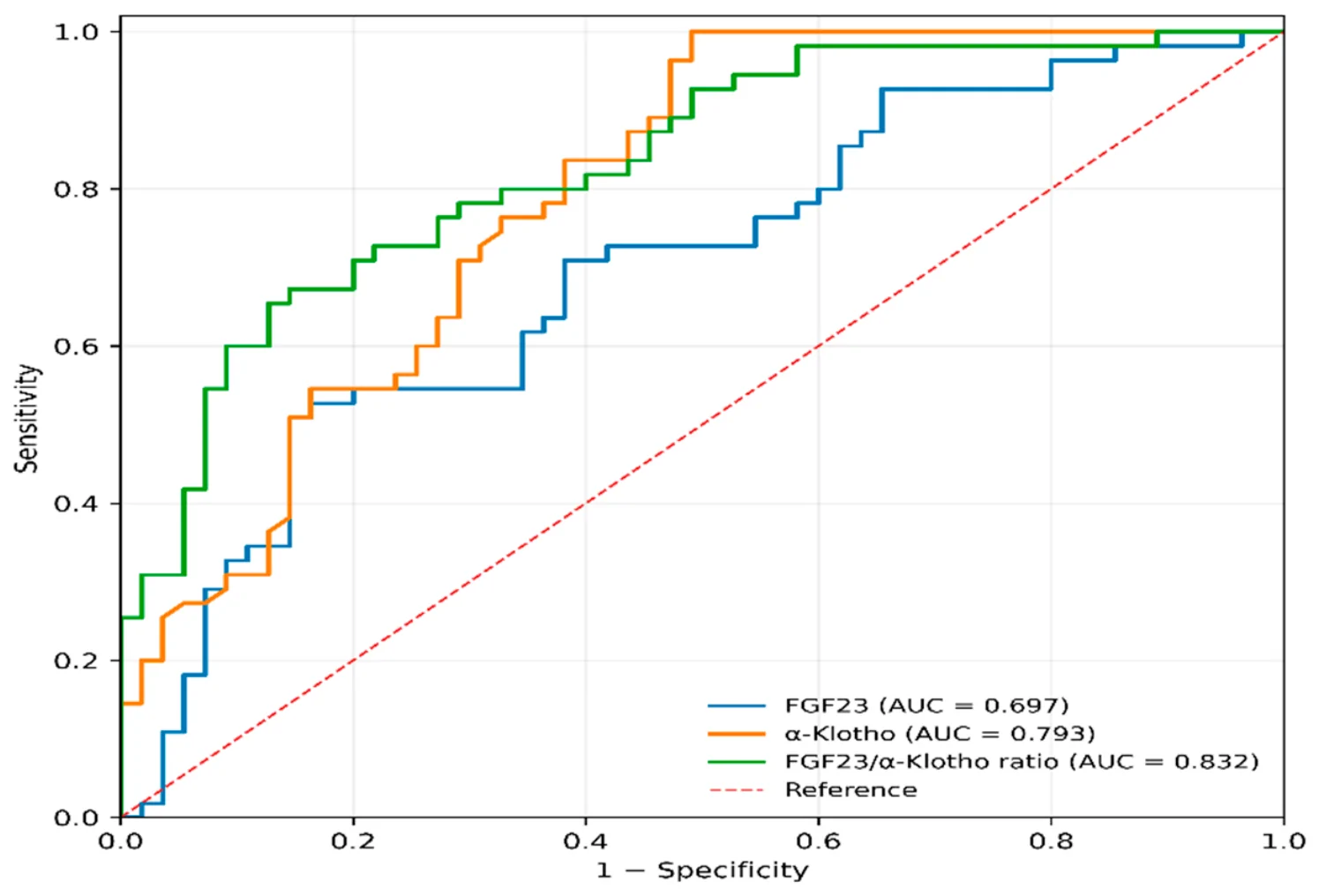
Receiver operating characteristic (ROC) curves for serum FGF23, α-Klotho, and the FGF23/α-Klotho ratio for identifying osteoporosis among postmenopausal women (n = 110). The FGF23/α-Klotho ratio demonstrated the highest discriminative performance (AUC = 0.832), followed by α-Klotho (AUC = 0.793) and FGF23 (AUC = 0.697).

The diagnostic performance of serum FGF23, α-Klotho, and the FGF23/α-Klotho ratio in postmenopausal women is summarized in Table 7 and graphically illustrated by the ROC curves in Figure 3. When the diagnostic analysis was restricted to postmenopausal women (n = 110), the FGF23/α-Klotho ratio demonstrated the highest discriminative performance for osteoporosis (AUC = 0.832, 95% bootstrap CI: 0.755–0.901), followed by α-Klotho (AUC = 0.793, 95% bootstrap CI: 0.708–0.869) and FGF23 (AUC = 0.697, 95% bootstrap CI: 0.600–0.795). Using the optimal cut-off value of 0.1624, the FGF23/α-Klotho ratio showed a sensitivity of 65.5% and a specificity of 87.3%.

Pairwise bootstrap comparisons of the ROC curves showed that the FGF23/α-Klotho ratio had significantly greater discriminative performance than FGF23 alone (ΔAUC = 0.135, *p* = 0.001). In contrast, the differences between α-Klotho and FGF23 (ΔAUC = 0.096, *p* = 0.171) and between the FGF23/α-Klotho ratio and α-Klotho (ΔAUC = 0.038, *p* = 0.270) were not statistically significant (Table 8). The FGF23/α-Klotho ratio therefore showed the highest numerical discriminative performance and significantly outperformed FGF23 alone; however, its discriminative performance was not significantly different from that of α-Klotho alone.

Formal paired bootstrap comparisons demonstrated that addition of FGF23 alone to the clinical reference model did not significantly increase discrimination (ΔAUC = 0.049, 95% CI: −0.013 to 0.116; *p* = 0.131). In contrast, addition of α-Klotho, the FGF23/α-Klotho ratio, or combined FGF23 and α-Klotho significantly increased the AUC by 0.114, 0.132, and 0.139, respectively (all *p* ≤ 0.004) (Table 9). For the final combined model compared with Model 2, the category-free NRI was 0.909 (95% bootstrap CI: 0.582–1.236; *p* < 0.001) and the IDI was 0.279 (95% bootstrap CI: 0.194–0.364; *p* < 0.001). The Brier score decreased from 0.206 to 0.140. Following bootstrap internal validation, the final model had an optimism-corrected AUC of 0.859, an optimism-corrected Brier score of 0.159, a calibration slope of 0.865, and a calibration intercept of 0.013. The apparent and bootstrap-corrected calibration of the final sequential model is shown in Supplementary Figure S1.

Because renal function may influence circulating FGF23 and α-Klotho concentrations, additional eGFR-adjusted diagnostic sensitivity analyses were performed. The reference model including age, BMI, 25(OH)D, and eGFR had an AUC of 0.785. Addition of FGF23, α-Klotho, or the FGF23/α-Klotho ratio increased the AUC to 0.830, 0.881, and 0.896, respectively, whereas simultaneous inclusion of FGF23 and α-Klotho resulted in the highest AUC of 0.907. Compared with the eGFR-adjusted reference model, the increases in AUC were 0.044 for FGF23 (95% bootstrap CI: 0.003–0.100; *p* = 0.028), 0.096 for α-Klotho (95% CI: 0.033–0.156; *p* < 0.001), 0.110 for the FGF23/α-Klotho ratio (95% CI: 0.039–0.170; *p* < 0.001), and 0.122 for the combined FGF23 and α-Klotho model (95% CI: 0.051–0.183; *p* < 0.001). These findings indicate that the discriminatory contribution of the FGF23–Klotho axis remained evident after accounting for renal function (Supplementary Table S2).

A sensitivity analysis was performed after excluding six postmenopausal women with a history of fragility fracture and T-scores > −2.5 at all evaluated skeletal sites. The principal findings remained materially unchanged. In the postmenopausal sensitivity cohort (n = 104; 49 non-osteoporotic and 55 osteoporotic women), the FGF23/α-Klotho ratio retained the highest discriminative performance for osteoporosis (AUC = 0.829, 95% bootstrap CI: 0.745–0.901), followed by α-Klotho (AUC = 0.781, 95% bootstrap CI: 0.691–0.866) and FGF23 (AUC = 0.699, 95% bootstrap CI: 0.592–0.794). The FGF23/α-Klotho ratio remained significantly superior to FGF23 alone (ΔAUC = 0.130, bootstrap *p* = 0.001), whereas the other pairwise differences were not statistically significant. Age- and BMI-adjusted correlations and multivariable regression findings also remained consistent with the primary analyses.

**Figure 4:**
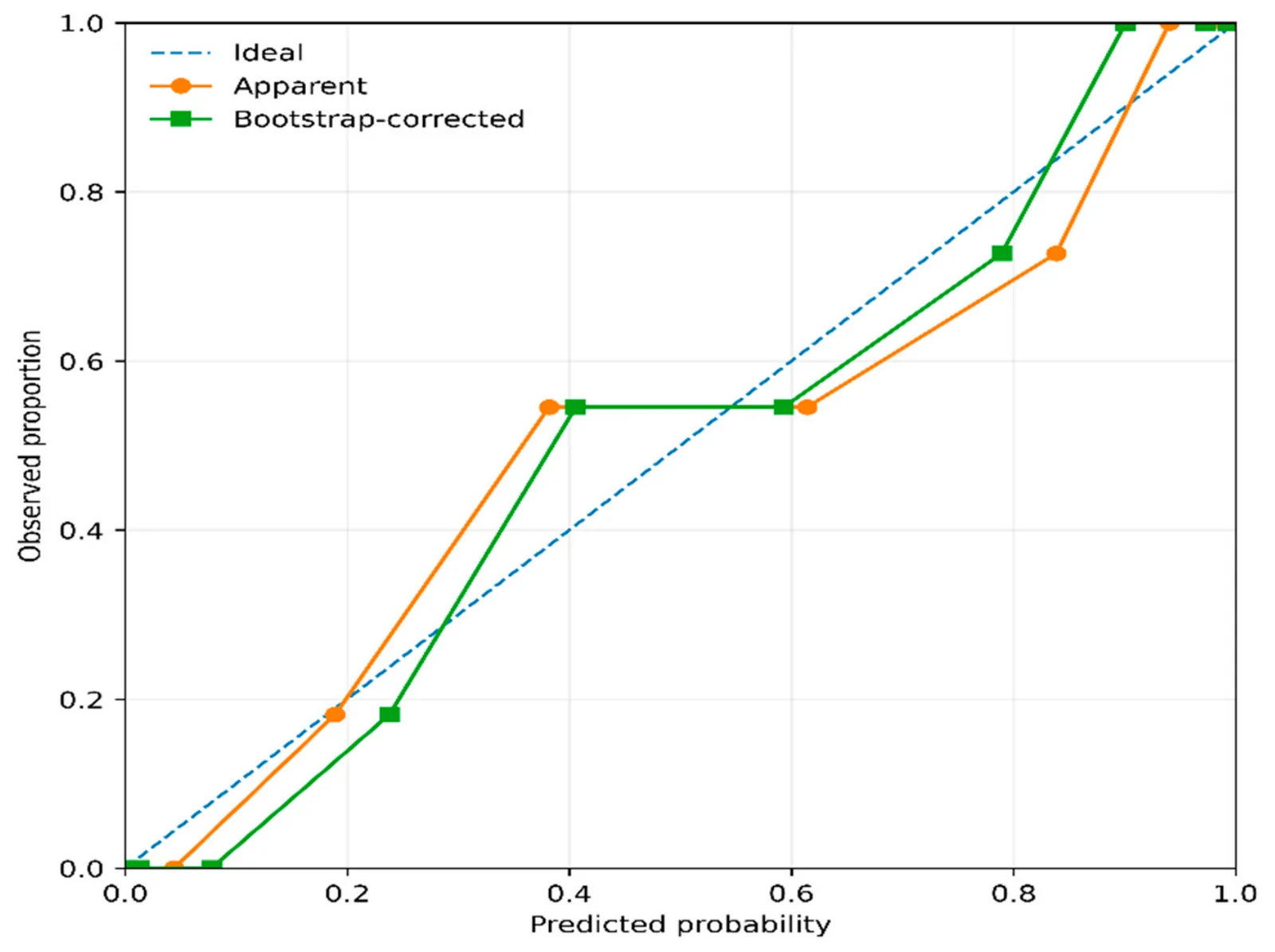
Calibration plot for the multivariable logistic regression model identifying postmenopausal osteoporosis. The dashed diagonal line represents perfect calibration. Apparent and bootstrap-corrected calibration curves are shown. Internal validation was performed using 2000 bootstrap resamples.

Bootstrap-based internal validation of the multivariable logistic regression model showed an apparent AUC of 0.952. The estimated optimism in AUC was 0.016, yielding an optimism-corrected AUC of 0.937. The apparent Brier score was 0.090, whereas the optimism-corrected Brier score was 0.113. The optimism-corrected calibration slope was 0.794, with a calibration-in-the-large intercept of 0.004. Thus, the model retained strong discrimination after internal validation, although the calibration slope indicated some degree of optimism in the fitted model (Figure 4).

## 4. Discussion

To the best of our knowledge, this is the first study to investigate the incremental diagnostic value of the FGF23/α-Klotho ratio beyond measured clinical variables for identifying PMOP. The present study demonstrated that serum FGF23 and α-Klotho are closely associated with bone mineral density and bone turnover in postmenopausal women. The principal finding was that the FGF23/α-Klotho ratio showed the strongest association with skeletal measures and the highest numerical discriminative performance among the evaluated biomarkers. Although the FGF23/α-Klotho ratio had the highest AUC, its discriminative performance was significantly greater than that of FGF23 alone but not significantly different from α-Klotho alone. Moreover, the addition of FGF23 and α-Klotho to the clinical reference model significantly increased discrimination, suggesting that the FGF23/α-Klotho axis provides additional discriminatory information beyond the measured clinical and biochemical variables. These findings support an association between alterations in the FGF23–Klotho axis and poorer skeletal status and highlight its potential utility as a complementary biomarker requiring further validation.

The observed differences in baseline demographic characteristics are consistent with the well-established epidemiology of postmenopausal osteoporosis. As expected, both postmenopausal groups were significantly older than the premenopausal healthy women, reflecting the physiological effects of ovarian aging on bone metabolism. Estrogen deficiency after menopause accelerates bone remodeling, increases osteoclast activity, and results in progressive bone loss, making age and menopausal status major determinants of osteoporosis risk [23,24]. Interestingly, although the two postmenopausal groups were of comparable age and had similar menopause duration, women with osteoporosis experienced menopause at a significantly younger age than those without osteoporosis. Earlier menopause has consistently been associated with lower peak skeletal reserve, prolonged exposure to estrogen deficiency, and an increased lifetime risk of osteoporosis and fragility fractures [25,26,27]. In contrast, lifestyle-related factors, including smoking, alcohol consumption, physical activity, and family history of osteoporosis, did not differ significantly among the study groups. These findings suggest that the differences observed in bone mineral density and circulating FGF23 and α-Klotho concentrations are unlikely to be explained by these baseline characteristics alone, thereby supporting the biological relevance of the observed biomarker alterations.

Postmenopausal women experience ongoing loss of bone mass, with resulting increases in the risk of fracture [28]. The marked differences in BMD observed across the three study groups confirm the validity of the study population and reflect the progressive skeletal deterioration associated with menopause and osteoporosis. As expected, premenopausal healthy women exhibited the highest BMD values at all measured skeletal sites, whereas postmenopausal women with osteoporosis had the lowest lumbar spine, femoral neck, and total hip BMD and T-scores. Furthermore, significant differences between the postmenopausal non-osteoporotic and osteoporotic groups indicate that bone loss progresses beyond the physiological decline associated with menopause alone. These findings are consistent with the established pathophysiology of postmenopausal osteoporosis, in which estrogen deficiency accelerates bone remodeling, resulting in an imbalance between bone formation and resorption and ultimately leading to progressive reductions in BMD [23,28]. Similar declines in lumbar spine and proximal femur BMD have been consistently reported in large epidemiological studies and constitute the basis for the WHO densitometric criterion for osteoporosis [24,29]. Therefore, the clear gradient in BMD across the study groups provides a robust framework for interpreting the observed alterations in circulating FGF23 and α-Klotho concentrations.

The biochemical findings observed in the present study are consistent with the altered mineral metabolism and accelerated bone turnover that characterize postmenopausal osteoporosis. Women with osteoporosis exhibited significantly higher serum phosphorus, PTH, ALP, P1NP, and β-CTX concentrations, accompanied by lower 25-hydroxyvitamin D levels, indicating increased skeletal remodeling and secondary disturbances in calcium–phosphate homeostasis. Elevated β-CTX and P1NP concentrations reflect enhanced bone resorption and compensatory bone formation, respectively, although the increase in bone resorption predominates after menopause, resulting in progressive bone loss [30,31]. Similarly, increased circulating PTH together with reduced vitamin D concentrations has been recognized as a common feature of postmenopausal osteoporosis and contributes to increased osteoclast activation and cortical bone loss [23,32,33]. In contrast, serum calcium and corrected calcium remained within a relatively narrow physiological range across all groups, highlighting the tight homeostatic regulation of circulating calcium despite substantial differences in bone metabolism. The modest reduction in eGFR observed in the osteoporotic group is also noteworthy, as renal function is closely involved in phosphate handling and the regulation of the FGF23–Klotho axis. Collectively, these biochemical alterations provide a biological context for the observed changes in circulating FGF23 and α-Klotho concentrations and support an association between dysregulation of mineral metabolism and osteoporosis. Importantly, the diagnostic contribution of the FGF23–Klotho axis remained evident after incorporation of eGFR into the clinical reference model. This finding suggests that the observed discriminatory performance is not attributable solely to differences in renal function, although residual renal-related confounding cannot be completely excluded.

One of the most important findings of the present study was the progressive increase in serum FGF23 concentrations and the concomitant decline in circulating α-Klotho levels across the spectrum from premenopausal healthy women to postmenopausal women with osteoporosis. Consequently, the FGF23/α-Klotho ratio increased markedly with worsening skeletal status, indicating an imbalance of the FGF23–Klotho axis across the observed spectrum of bone health. These findings are biologically plausible, as FGF23 requires α-Klotho as an essential co-receptor to regulate phosphate and vitamin D metabolism, and disruption of this endocrine axis has been implicated in impaired bone remodeling and skeletal fragility [11,15,34]. Experimental studies have shown that Klotho deficiency enhances FGF23 resistance, alters phosphate homeostasis, and accelerates age-related skeletal changes, whereas elevated circulating FGF23 has been associated with adverse bone outcomes in several clinical settings [11,13,35,36,37,38]. Although previous studies investigating FGF23 or α-Klotho individually in osteoporosis have yielded inconsistent results, our findings suggest that simultaneous evaluation of both biomarkers, particularly through the FGF23/α-Klotho ratio, may provide complementary information regarding alterations in mineral metabolism. The FGF23/α-Klotho ratio should, however, be interpreted as an exploratory composite index rather than a direct stoichiometric measure of FGF23–Klotho signaling. Although its biological rationale is supported by the functional dependence of FGF23 signaling on α-Klotho, the two analytes were quantified using different ELISA systems. Consequently, differences in assay calibration, analytical imprecision, matrix effects, and between-assay comparability may influence the absolute value of the ratio. Ratio-derived cut-offs may therefore be assay-dependent and should not be considered universally transferable without external analytical and clinical validation. Importantly, the principal findings did not rely exclusively on the ratio: models including FGF23 and α-Klotho simultaneously as separate variables also demonstrated substantial discriminatory performance, supporting the relevance of the joint FGF23–Klotho axis while avoiding dependence on a simple concentration ratio alone.

The correlation analyses further demonstrate consistent associations between the FGF23–Klotho axis and postmenopausal bone metabolism. Serum FGF23 was moderately and consistently associated with lower BMD at all skeletal sites, whereas α-Klotho exhibited positive correlations with both BMD and T-scores. Notably, FGF23 was positively correlated with markers of bone turnover, including ALP, P1NP, osteocalcin, and β-CTX, as well as serum phosphorus and PTH, while showing inverse associations with 25-hydroxyvitamin D and eGFR. Conversely, α-Klotho demonstrated the opposite correlation profile. These findings are biologically plausible because FGF23 regulates phosphate excretion and suppresses renal 1α-hydroxylase activity, thereby reducing active vitamin D synthesis, whereas α-Klotho serves as the essential co-receptor for FGF23 signaling and exerts additional protective effects on bone and mineral metabolism [39,40,41,42]. Experimental studies have demonstrated that disruption of the FGF23–Klotho axis results in abnormalities of phosphate homeostasis, impaired bone mineralization, and altered skeletal remodeling [11,34,39,40,41,42,43]. The observed association between elevated FGF23 and increased β-CTX and P1NP further suggests that higher FGF23 concentrations are associated with greater bone turnover in postmenopausal women. Likewise, the inverse correlations between α-Klotho and bone turnover markers support previous evidence indicating that reduced Klotho availability is associated with skeletal aging and osteoporosis [4,11,16,36,42,43].

Importantly, the correlation coefficients were of moderate magnitude (|r| ≈ 0.45–0.51 for BMD), suggesting that although the FGF23–Klotho axis is closely related to bone health, osteoporosis remains a multifactorial disease influenced by hormonal, metabolic, genetic, and lifestyle-related factors. Nevertheless, the consistent and opposite correlation patterns observed for FGF23 and α-Klotho across bone density, bone turnover, and mineral metabolism parameters strengthen the biological relevance of this endocrine axis in postmenopausal osteoporosis.

The multivariable regression analyses further demonstrated associations between the FGF23–Klotho axis and skeletal status in postmenopausal women. After adjustment for established determinants of bone metabolism, including age, BMI, PTH, 25-hydroxyvitamin D, and renal function, serum FGF23 remained independently and inversely associated with lumbar spine, femoral neck, and total hip BMD, whereas α-Klotho was independently associated with higher BMD at all skeletal sites. Notably, the FGF23/α-Klotho ratio demonstrated the strongest independent association with bone mineral density, yielding the largest standardized regression coefficients and the highest adjusted R^2^ values. Furthermore, multivariable logistic regression identified elevated FGF23 and reduced α-Klotho concentrations as independent factors associated with postmenopausal osteoporosis, even after adjustment for conventional clinical and biochemical risk factors. The persistence of these associations after multivariable adjustment suggests that alterations in the FGF23–Klotho axis remained associated with poorer skeletal status after adjustment for age, adiposity, vitamin D status, PTH, and renal function. These findings are biologically plausible because FGF23 and α-Klotho jointly regulate phosphate homeostasis, vitamin D metabolism, and osteocyte function, all of which are essential for maintaining bone quality and mineralization [11,34,44]. Importantly, our results indicate that evaluating the functional interaction between FGF23 and α-Klotho, rather than either biomarker alone, may provide a more comprehensive assessment of osteoporosis-related alterations in mineral metabolism.

The observation that age showed an inverse association with osteoporosis, whereas BMI was not independently associated in the adjusted logistic regression model, should be interpreted cautiously. Although both variables are recognized determinants of fracture risk in epidemiological studies, their associations in the present model may have been influenced by the restricted age distribution of the postmenopausal cohort and adjustment for correlated biochemical variables. In contrast, the independent associations of FGF23 and α-Klotho persisted, suggesting that these biomarkers may reflect biological alterations associated with postmenopausal osteoporosis after adjustment for the measured clinical and biochemical covariates. The diagnostic analyses further highlight the discriminatory performance of the FGF23–Klotho axis in postmenopausal osteoporosis. Among the evaluated biomarkers, the FGF23/α-Klotho ratio demonstrated the highest numerical discriminative performance, followed by α-Klotho and FGF23. Pairwise comparison of ROC curves confirmed that the FGF23/α-Klotho ratio significantly outperformed FGF23 alone, whereas its discriminative ability was not significantly different from that of α-Klotho. These findings suggest that incorporating both components of the FGF23–Klotho signaling pathway provides a more comprehensive assessment of osteoporosis-related alterations than measuring FGF23 in isolation. Because FGF23 requires α-Klotho as an obligate co-receptor for biological activity, the ratio may better reflect the functional status of this endocrine axis than either biomarker alone. This concept is supported by experimental studies demonstrating that disruption of the FGF23–Klotho axis impairs phosphate homeostasis, suppresses active vitamin D synthesis, and adversely affects bone remodeling and mineralization [11,45,46,47,48,49,50].

An additional strength of the present study is the evaluation of the incremental diagnostic value of these biomarkers beyond conventional clinical risk factors. Sequential ROC analyses demonstrated that the clinical reference model including age, BMI, and 25-hydroxyvitamin D had an AUC of 0.743. Addition of FGF23 increased the AUC to 0.792, although this increase was not statistically significant. In contrast, addition of α-Klotho or the FGF23/α-Klotho ratio increased the AUC to 0.857 and 0.875, respectively, while the combined model incorporating both FGF23 and α-Klotho achieved the highest AUC of 0.882. Formal paired bootstrap comparisons demonstrated significant incremental discrimination for α-Klotho, the FGF23/α-Klotho ratio, and the combined FGF23–α-Klotho model relative to the clinical reference model. These findings indicate that circulating FGF23 and α-Klotho may provide additional discriminatory information beyond the measured clinical variables. Although previous studies have mainly focused on the individual associations of FGF23 or α-Klotho with bone metabolism, few have examined their combined diagnostic utility. Our results therefore suggest that simultaneous assessment of these biomarkers warrants further evaluation as a potential adjunct to current osteoporosis assessment strategies. Nevertheless, external validation in larger, prospective, multicenter cohorts is warranted before incorporation of these biomarkers into routine clinical practice.

Taken together, these findings support an association between the FGF23–Klotho axis and skeletal health in postmenopausal women; however, the cross-sectional design does not establish whether alterations in these biomarkers precede, result from, or directly contribute to osteoporosis.

### 4.1. Strengths and Limitations

This study has several limitations. First, its cross-sectional, single-center design precludes establishing causal relationships between the FGF23–Klotho axis and osteoporosis and may limit the generalizability of the findings to other populations. Second, although comprehensive multivariable analyses were performed, residual confounding from unmeasured factors remains possible. Although age, BMI, 25-hydroxyvitamin D, PTH, and renal function were considered in the adjusted analyses, other determinants of mineral metabolism and skeletal health, including dietary calcium and phosphate intake, calcium and vitamin D supplementation, sunlight exposure, estrogen status and other sex hormone concentrations, parathyroid-related factors not captured by a single PTH measurement, genetic predisposition, and detailed concomitant medication use, were not comprehensively assessed. These unmeasured factors may have influenced both circulating FGF23 and α-Klotho concentrations and skeletal outcomes; therefore, the observed associations cannot be considered fully independent of residual confounding. Third, circulating FGF23 and α-Klotho concentrations were measured at a single time point; therefore, temporal changes in these biomarkers during disease progression or treatment could not be evaluated. In addition, characteristics of eligible women who declined participation were not systematically recorded; therefore, potential non-participation-related selection bias cannot be excluded. Finally, prospective incident fracture outcomes and longitudinal follow-up data were not available, preventing assessment of the prognostic value of these biomarkers for subsequent osteoporotic fractures. Group allocation was based on the prespecified DXA-based operational definition; therefore, six postmenopausal women with a history of fragility fracture and T-scores > −2.5 at all evaluated skeletal sites were classified as non-osteoporotic. Although this classification may have introduced some degree of clinical misclassification, exclusion of these participants in a sensitivity analysis did not materially alter the principal findings. Bootstrap internal validation further demonstrated that discrimination remained high after correction for optimism; however, the optimism-corrected calibration slope of 0.794 indicated some degree of model overfitting. Similarly, for the sequential diagnostic model, the reduction from an apparent AUC of 0.882 to an optimism-corrected AUC of 0.859 indicates some model optimism despite favorable incremental discrimination and reclassification measures. Therefore, the multivariable and incremental diagnostic models should be considered exploratory until externally validated in larger independent cohorts.

Despite these limitations, the present study has several important strengths. To our knowledge, this is among the few studies to simultaneously evaluate serum FGF23, α-Klotho, and the FGF23/α-Klotho ratio in relation to bone mineral density, bone turnover markers, and the diagnostic performance for postmenopausal osteoporosis. Another major strength is the comprehensive statistical approach, including age- and BMI-adjusted partial correlation analyses, multivariable linear and logistic regression models, ROC curve analyses, pairwise bootstrap comparisons of ROC curves, and incremental diagnostic models, which enabled evaluation of both the independent associations and the additional diagnostic value of these biomarkers beyond conventional clinical risk factors. The inclusion of three well-defined study groups representing different stages of bone health further strengthened the ability to characterize progressive alterations in the FGF23–Klotho axis across the spectrum from normal bone metabolism to established osteoporosis.

### 4.2. Clinical Implications

The present findings indicate that assessment of the FGF23–Klotho axis may complement, rather than replace, current osteoporosis evaluation strategies. The FGF23/α-Klotho ratio showed the highest numerical discriminative performance among the evaluated biomarkers, although its performance was not significantly different from that of α-Klotho alone. Models incorporating α-Klotho, the FGF23/α-Klotho ratio, or FGF23 and α-Klotho jointly provided additional discriminatory information beyond the measured clinical variables. If validated in larger prospective cohorts, incorporation of FGF23 and α-Klotho into multivariable diagnostic models may potentially complement individualized osteoporosis assessment alongside established tools such as DXA and conventional biochemical markers.

## 5. Conclusions

In conclusion, alterations in the FGF23–Klotho axis were closely associated with reduced bone mineral density and altered bone metabolism in postmenopausal women. The FGF23/α-Klotho ratio showed the highest numerical discriminative performance among the evaluated biomarkers, although its performance was not significantly different from that of α-Klotho alone. The combined assessment of FGF23 and α-Klotho provided additional discriminatory information beyond the measured conventional clinical variables. Together with the independent associations identified in multivariable analyses, these findings suggest that assessment of the FGF23–Klotho axis may complement current approaches to osteoporosis risk evaluation. However, given the cross-sectional design, potential residual confounding, and lack of external validation, these findings should be considered exploratory. Future large-scale prospective studies are warranted to validate these observations, establish clinically applicable cut-off values, and determine whether incorporation of these biomarkers improves fracture risk prediction and clinical decision-making. 

## Figures and Tables

**Table 1:** Baseline demographic characteristics according to study groups.

Variable	Premenopausal Healthy (n = 55)	Postmenopausal Non-Osteoporotic (n = 55)	Postmenopausal Osteoporotic (n = 55)	*p*
Age (years)	45.07 ± 3.42	55.82 ± 3.20	55.27 ± 2.68	<0.001
Body mass index (kg/m^2^)	25.76 ± 4.00	28.40 ± 4.36	27.47 ± 5.70	0.014
Menopause age (years) †	—	48.31 ± 2.64	47.33 ± 2.41	0.044
Menopause duration (years) †	—	7.51 ± 3.57	7.95 ± 3.33	0.509
Current smoker, n (%)	8 (14.5)	9 (16.4)	11 (20.0)	0.740
Alcohol consumption, n (%)	0 (0.0)	4 (7.3)	1 (1.8)	0.068
Physical activity, n (%)				0.776
High	7 (12.7)	8 (14.5)	10 (18.2)	
Moderate	25 (45.5)	26 (47.3)	20 (36.4)	
Sedentary	23 (41.8)	21 (38.2)	25 (45.5)	
Family history of osteoporosis, n (%)	8 (14.5)	10 (18.2)	14 (25.5)	0.338

Data are presented as mean ± standard deviation (SD) or n (%). Continuous variables were compared using one-way ANOVA, except menopause-related variables, which were compared only between the two postmenopausal groups using the independent samples *t*-test. Categorical variables were compared using the χ^2^ test. † Applicable only to postmenopausal women.

**Table 2:** Biochemical parameters according to study groups.

Variable	Premenopausal Healthy (n = 55)	Postmenopausal Non-Osteoporotic (n = 55)	Postmenopausal Osteoporotic (n = 55)	*p*
Calcium (mg/dL)	9.37 ± 0.38	9.41 ± 0.39	9.39 ± 0.28	0.884
Corrected calcium (mg/dL)	9.16 ± 0.46	9.21 ± 0.43	9.19 ± 0.40	0.825
Phosphorus (mg/dL)	3.35 ± 0.36 ^a^	3.47 ± 0.35 ^ab^	3.59 ± 0.33 ^b^	0.002
Magnesium (mg/dL)	2.03 ± 0.21	2.05 ± 0.16	2.07 ± 0.17	0.556
ALP (U/L)	75.02 ± 17.74 ^a^	82.48 ± 23.51 ^a^	104.86 ± 29.47 ^b^	<0.001
Bone-specific ALP (µg/L)	27.74 ± 10.16 ^a^	30.66 ± 12.70 ^a^	39.09 ± 13.73 ^b^	<0.001
Parathyroid hormone (pg/mL)	38.01 ± 13.01 ^a^	42.99 ± 16.45 ^a^	64.46 ± 19.57 ^b^	<0.001
25-Hydroxyvitamin D (ng/mL)	26.66 ± 8.65 ^a^	21.33 ± 6.49 ^b^	16.13 ± 5.58 ^c^	<0.001
Osteocalcin (ng/mL)	23.66 ± 4.77 ^a^	24.93 ± 5.11 ^a^	28.45 ± 4.66 ^b^	<0.001
P1NP (ng/mL)	53.64 ± 15.53 ^a^	59.61 ± 15.07 ^a^	73.06 ± 13.61 ^b^	<0.001
β-CTX (ng/mL)	0.31 ± 0.09 ^a^	0.45 ± 0.13 ^b^	0.57 ± 0.19 ^c^	<0.001
Urea (mg/dL)	32.07 ± 6.87	34.32 ± 7.33	34.64 ± 7.96	0.143
Creatinine (mg/dL)	0.75 ± 0.11	0.78 ± 0.12	0.80 ± 0.11	0.063
eGFR (mL/min/1.73 m^2^)	84.71 ± 11.73 ^a^	78.72 ± 10.06 ^a^	75.64 ± 8.36 ^b^	<0.001
C-reactive protein (mg/L)	2.22 ± 1.23 ^a^	3.12 ± 1.82 ^b^	3.56 ± 1.80 ^b^	<0.001

Different superscript letters (a, b, and c) indicate statistically significant differences between groups according to Tukey’s honestly significant difference (HSD) post hoc test (*p* < 0.05). Values sharing at least one common superscript letter are not significantly different.

**Table 3:** Spearman correlation analysis of serum FGF23 and α-Klotho with bone mineral density and biochemical parameters.

Variable	FGF23 (r)	*p*	α-Klotho (r)	*p*
Lumbar spine BMD (g/cm^2^)	−0.487	<0.001	0.452	<0.001
Lumbar spine T-score	−0.503	<0.001	0.469	<0.001
Femoral neck BMD (g/cm^2^)	−0.503	<0.001	0.481	<0.001
Femoral neck T-score	−0.492	<0.001	0.474	<0.001
Total hip BMD (g/cm^2^)	−0.499	<0.001	0.465	<0.001
Total hip T-score	−0.509	<0.001	0.472	<0.001
Phosphorus (mg/dL)	0.277	<0.001	−0.243	0.002
ALP (U/L)	0.260	0.001	−0.268	0.001
Bone-specific ALP (µg/L)	0.163	0.036	−0.191	0.014
Parathyroid hormone (pg/mL)	0.274	<0.001	−0.281	<0.001
25-Hydroxyvitamin D (ng/mL)	−0.255	0.001	0.238	0.002
Osteocalcin (ng/mL)	0.232	0.003	−0.214	0.006
P1NP (ng/mL)	0.249	0.001	−0.227	0.003
β-CTX (ng/mL)	0.361	<0.001	−0.332	<0.001
eGFR (mL/min/1.73 m^2^)	−0.184	0.018	0.176	0.024

Data are presented as Spearman’s correlation coefficients (r). BMD, bone mineral density; ALP, alkaline phosphatase; P1NP, procollagen type I N-terminal propeptide; β-CTX, beta C-terminal telopeptide of type I collagen; eGFR, estimated glomerular filtration rate.

**Table 4:** Age- and BMI-adjusted partial Spearman correlation analysis between serum FGF23, α-Klotho, the FGF23/α-Klotho ratio, and bone mineral density T-scores.

Variable	Lumbar Spine T-Score ρ (*p*)	Femoral Neck T-Score ρ (*p*)	Total Hip T-Score ρ (*p*)
FGF23	−0.326 (<0.001)	−0.327 (<0.001)	−0.346 (<0.001)
α-Klotho	0.522 (<0.001)	0.455 (<0.001)	0.496 (<0.001)
FGF23/α-Klotho ratio	−0.544 (<0.001)	−0.504 (<0.001)	−0.533 (<0.001)

Data are presented as age- and body mass index (BMI)-adjusted partial Spearman correlation coefficients (ρ).

**Table 5:** Multivariable linear regression analyses of the associations of serum FGF23, α-Klotho, and the FGF23/α-Klotho ratio with bone mineral density T-scores.

Outcome	Biomarker Model	B	SE	β	*p*	Adjusted R^2^
Lumbar spine T-score	FGF23	−0.0016	0.0004	−0.247	<0.001	0.525
Lumbar spine T-score	α-Klotho	0.0003	0.00004	0.380	<0.001	0.589
Lumbar spine T-score	FGF23/α-Klotho ratio	−6.150	0.867	−0.404	<0.001	0.602
Femoral neck T-score	FGF23	−0.0014	0.0004	−0.238	<0.001	0.481
Femoral neck T-score	α-Klotho	0.0002	0.00004	0.327	<0.001	0.520
Femoral neck T-score	FGF23/α-Klotho ratio	−5.018	0.804	−0.379	<0.001	0.548
Total hip T-score	FGF23	−0.0012	0.0003	−0.227	<0.001	0.506
Total hip T-score	α-Klotho	0.0002	0.00003	0.333	<0.001	0.552
Total hip T-score	FGF23/α-Klotho ratio	−4.686	0.688	−0.396	<0.001	0.586

Each biomarker was entered into a separate multivariable linear regression model adjusted for age, body mass index (BMI), parathyroid hormone (PTH), 25-hydroxyvitamin D, and estimated glomerular filtration rate (eGFR). Standardized regression coefficients (β) are presented.

**Table 6:** Multivariable logistic regression analysis of factors independently associated with postmenopausal osteoporosis among postmenopausal women (n = 110).

Variable	OR	95% CI	*p*
Age (years)	0.72	0.53–0.96	0.028
BMI (kg/m^2^)	0.93	0.81–1.08	0.354
25(OH) Vitamin D (ng/mL)	0.83	0.73–0.94	0.003
PTH (pg/mL)	1.11	1.05–1.16	<0.001
FGF23 (pg/mL)	1.007	1.003–1.012	0.002
α-Klotho (pg/mL)	0.999	0.998–0.999	<0.001

Model statistics: Nagelkerke R^2^ = 0.759; likelihood ratio test, *p* < 0.001. The analysis was restricted to postmenopausal women (n = 110; 55 non-osteoporotic and 55 osteoporotic women). The multivariable model included age, BMI, 25-hydroxyvitamin D, PTH, FGF23, and α-Klotho. ORs represent the change in the odds of osteoporosis for each one-unit increase in the corresponding variable. OR, odds ratio; CI, confidence interval; BMI, body mass index; PTH, parathyroid hormone; FGF23, fibroblast growth factor 23.

**Table 7:** Diagnostic performance of serum FGF23, α-Klotho, and the FGF23/α-Klotho ratio for identifying osteoporosis among postmenopausal women (n = 110).

Parameter	AUC	95% CI	Optimal Cut-Off	Sensitivity (%)	Specificity (%)
FGF23 (pg/mL)	0.697	0.600–0.795	720	50.9	85.5
α-Klotho (pg/mL)	0.793	0.708–0.869	5680	100.0	50.9
FGF23/α-Klotho ratio	0.832	0.755–0.901	0.1624	65.5	87.3

ROC analyses were restricted to postmenopausal women (n = 110; 55 non-osteoporotic and 55 osteoporotic women). Optimal cut-off values were determined using the Youden index. AUC, area under the receiver operating characteristic curve; CI, confidence interval; FGF23, fibroblast growth factor 23; ROC, receiver operating characteristic.

**Table 8:** Pairwise comparison of ROC curve AUCs in postmenopausal women.

Comparison	ΔAUC	Bootstrap *p* Value	Interpretation
FGF23 vs. α-Klotho	0.096	0.171	Not significant
FGF23 vs. FGF23/α-Klotho ratio	0.135	0.001	Significant
α-Klotho vs. FGF23/α-Klotho ratio	0.038	0.270	Not significant

AUC comparisons were performed using paired bootstrap resampling with 2000 iterations.

**Table 9:** Incremental diagnostic performance and formal comparison of sequential models for identifying osteoporosis among postmenopausal women.

Model	Variables	AUC (95% Bootstrap CI)	ΔAUC vs. Model 2 (95% CI)	Bootstrap *p*
Model 1	Age + BMI	0.589 (0.480–0.694)	—	—
Model 2	Age + BMI + 25(OH)D	0.743 (0.653–0.836)	Reference	—
Model 3	Model 2 + FGF23	0.792 (0.706–0.878)	0.049 (−0.013–0.116)	0.131
Model 4	Model 2 + α-Klotho	0.857 (0.788–0.919)	0.114 (0.039–0.194)	0.004
Model 5	Model 2 + FGF23/α-Klotho ratio	0.875 (0.808–0.934)	0.132 (0.050–0.215)	0.001
Model 6	Model 2 + FGF23 + α-Klotho	0.882 (0.819–0.937)	0.139 (0.058–0.222)	<0.001

ROC analyses were restricted to postmenopausal women (n = 110; 55 non-osteoporotic and 55 osteoporotic women). Model 2 served as the clinical reference model. Differences in AUC were assessed using paired bootstrap resampling with 2000 iterations. AUC, area under the receiver operating characteristic curve; BMI, body mass index; FGF23, fibroblast growth factor 23; 25(OH)D, 25-hydroxyvitamin D.

## Data Availability

The data that support the findings of this study are available on request from the corresponding author. The data are not publicly available due to privacy or ethical restrictions.

## Supplementary Materials

The following supporting information can be downloaded at: https://www.mdpi.com/article/10.3390/jcm15176716/s1, Figure S1: Calibration plot of the final sequential diagnostic model for identifying osteoporosis among postmenopausal women. The final model included age, BMI, 25-hydroxyvitamin D, FGF23, and α-Klotho. The dashed diagonal line represents perfect calibration. Apparent and bootstrap-corrected calibration curves are shown. Internal validation was performed using 2000 bootstrap resamples; Table S1: Bone mineral density (DXA) parameters according to study groups; Table S2: eGFR-adjusted sensitivity analysis of incremental diagnostic models for postmenopausal osteoporosis.
