# Supplementary material for: The FGF23/α-Klotho Axis in Postmenopausal Osteoporosis: Associations with Bone Mineral Density and Diagnostic Discrimination
Source: J Clin Med. 2026 Aug 29;15(17):6716. doi: 10.3390/jcm15176716 (PMC13566240; doi:10.3390/jcm15176716)
Supplement: Supplementary file 1 [file jcm-15-06716-s001.zip › jcm-4498852-supplementary.pdf]

Table S1. Bone mineral density (DXA) parameters according to study groups.

Variable	Premenopausal Healthy (n = 55)	Postmenopausal Non-Osteoporotic (n = 55)	Postmenopausal Osteoporotic (n = 55)	*P*
**Lumbar spine BMD (g/cm²)**	0.954 ± 0.053[a]	0.875 ± 0.059[b]	0.697 ± 0.034[c]	<0.001
**Lumbar spine T-score**	−0.455 ± 0.499[a]	−1.254 ± 0.557[b]	−3.013 ± 0.298[c]	<0.001
**Femoral neck BMD (g/cm²)**	0.808 ± 0.030[a]	0.764 ± 0.038[b]	0.642 ± 0.027[c]	<0.001
**Femoral neck T-score**	−0.533 ± 0.401[a]	−1.168 ± 0.493[b]	−2.735 ± 0.322[c]	<0.001
**Total hip BMD (g/cm²)**	0.907 ± 0.037[a]	0.848 ± 0.043[b]	0.753 ± 0.038[c]	<0.001
**Total hip T-score**	−0.306 ± 0.451[a]	−1.047 ± 0.514[b]	−2.199 ± 0.425[c]	<0.001

Data are presented as mean ± standard deviation (SD). Comparisons among groups were performed using one-way analysis of variance (ANOVA), followed by Tukey's honestly significant difference (HSD) post hoc test. Different superscript letters (a–c) indicate statistically significant differences between groups (adjusted $p < 0.05$). BMD, bone mineral density; DXA, dual-energy X-ray absorptiometry.

Supplementary Table S2. eGFR-adjusted sensitivity analysis of incremental diagnostic models for postmenopausal osteoporosis.

Model	Variables	AUC	ΔAUC vs. reference (95% bootstrap CI)	*p*
**Reference**	Age + BMI + 25(OH)D + eGFR	0.785	—	—
**+ FGF23**	Reference + FGF23	0.830	0.044 (0.003–0.100)	0.028
**+ α-Klotho**	Reference + α-Klotho	0.881	0.096 (0.033–0.156)	<0.001
**+ Ratio**	Reference + FGF23/α-Klotho ratio	0.896	0.110 (0.039–0.170)	<0.001
**+ FGF23 + α-Klotho**	Reference + FGF23 + α-Klotho	0.907	0.122 (0.051–0.183)	<0.001

ROC analyses were restricted to postmenopausal women (n = 110; 55 non-osteoporotic and 55 osteoporotic women). The reference model included age, BMI, 25-hydroxyvitamin D, and eGFR. Differences in AUC relative to the reference model were evaluated using paired bootstrap resampling with 2,000 iterations, and 95% bootstrap confidence intervals were calculated. AUC, area under the receiver operating characteristic curve; BMI, body mass index; 25(OH)D, 25-hydroxyvitamin D; eGFR, estimated glomerular filtration rate; FGF23, fibroblast growth factor 23; CI, confidence interval.

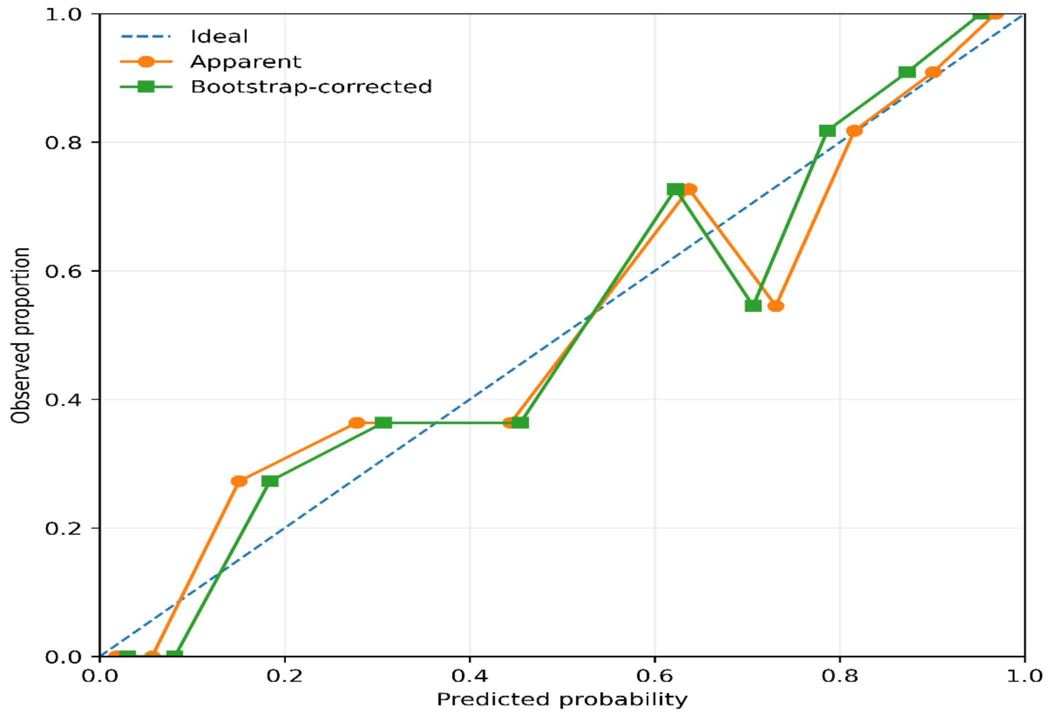

Supplementary Figure S1. Calibration plot of the final sequential diagnostic model for identifying osteoporosis among postmenopausal women. The final model included age, BMI, 25-hydroxyvitamin D, FGF23, and α-Klotho. The dashed diagonal line represents perfect calibration. Apparent and bootstrap-corrected calibration curves are shown. Internal validation was performed using 2,000 bootstrap resamples.
